# Practical Departments

**Published:** 1894-05-12

**Authors:** 


### PRACTICAL DEPARTMENTS.

### COMBINED BACK AND FOOT REST.

Under this heading in The Hospital for April 21st a sketch and description was given of a bed rest, with foot support. Our readers may like to know that rests of this kind can be obtained from Messrs. Mayer and Meltzer, 71, Great Portland Street, W., designed by Mr. Newton H. Nixon.

